# Temperature Dependency of Proton Pumping Activity for Marine Microbial Rhodopsin from Antartic Ocean

**DOI:** 10.1038/s41598-020-58023-5

**Published:** 2020-01-28

**Authors:** Se-Hwan Kim, ByungHoon Jung, Soon Gyu Hong, Kwang-Hwan Jung

**Affiliations:** 1 Department of Life Science and Institute of Biological Interfaces, Sogang University, 35 Baekbeom-Ro, Mapo-Gu, Seoul 04107 Korea; 2 Korea Polar Research Institute (KOPRI), 26 Songdomirae-ro, Yeonsu-gu, Incheon 21990 Korea; 3 GREEN CROSS CORP., 107, Ihyeon-ro 30beon-gil, Giheung-gu, Yongin-si, Gyeonggi-do 16924 Korea

**Keywords:** Biochemistry, Microbiology

## Abstract

Proteorhodopsin (PR) is discovered from marine bacteria and it has proton pumping activity from inside to outside of the cell using light energy. In general, PR classified into two groups by the maximum absorption spectra. In this study, we isolated the two of a full sequence of opsin homologues by PCR from the seawater sample near King George Island, Antarctica. One was the same sequence as the first reported GPR (Green-light absorbing PR) from Monterey Bay. Another named HSG119 was a newly discovered sequence which shows high sequence similarity with BPR (Blue-light absorbing PR). HSG119 has an absorption maximum at 493 nm with broader spectrum at pH7.0 and it can pump protons out of the cell membrane. Interestingly, it showed a similar temperature dependence to GPR(Y200N) that isolated near the North pole.

## Introduction

Rhodopsins are a light-harvesting protein that contains retinal as a chromophore and have seven trans-membrane alpha-helices. Their functions are ion pumping across a membrane, send a signal into the cell by light sensing, and channel for cation and anion. They were classified into type I and type II rhodopsins. The latter type is very famous for photosensitive receptor proteins in animal eyes that are human rod and cone cells, as we have known. They are not only existed in human but also fishes, frogs, lizards, birds and dogs. The former type and archaeal-type rhodopsin are well studied in *Halobacterium salinarum* halophilic archaea. The first discovered rhodopsins are bacteriorhodopsin (BR), halorhodopsin (HR), sensory rhodopsin I and II (SRI and SRII) in *H*. *salinarium*^1^. Those rhodopsins are categorized into type I rhodopsin. And type I rhodopsin has been found also in Archaea, Eubacteria and unicellular Eukarya^2–4^. That is the reason why type I is also called microbial rhodopsin. The big differences between type I and type II rhodopsin are the photoisomerization mechanism of the chromophore. While it is activated by light, photoisomerization process of type I rhodopsin occurs as retinal changes from all-trans to 13-cis form. On the other hand, retinal photoisomerization of type II rhodopsin is changed from 11-cis to all-trans retinal^5^.

Type I rhodopsins have been well studied as a model system, which makes a chemical gradient from light energy or contributed to phototaxis of host cell^6^. The first PR (proteorhodopsin) was discovered from uncultured marine gammaproteobacteria SAR86 group^7^. PR homologues were found in the Antarctic Ocean, Hawaii Ocean, Monterey Bay, Mediterranean Sea, the RED Sea and Sargasso Sea^8–11^. Most of PRs were discovered by PCR based gene survey, BAC and fosmid library screening, and environmental shotgun libraries^7,8,10,12–14^. PR works for light-driven proton pumping and has two important residues for its function^15–17^. One is Asp97 as proton acceptor and another is Glu108 as proton donor against the chromophore Schiff base in GPR (Green-light absorbing ProteoRhodopsin). In another type I rhodopsin, bacteriorhodopsin (BR), Asp85 and Asp96 work similar function, respectively^16,18^. PR easily produces M intermediate at the pH which higher than pKa value of proton acceptor and shows a proton transfer activity. On the other hand, in the case of lower pH than pKa value, M intermediate formed very slowly because the retinal Schiff base is not easy to be deprotonated^17–19^.

PR variants could be sorted into two groups mainly depending on their absorption maxima. One is green-light absorbing proteorhodopsin (GPR) and another is blue-light absorbing proteorhodopsin (BPR). The absorption maximum peak of PRs shows variability depends on the places where their host is living. The HOT_0m from Central North Pacific Ocean have green absorption maxima (525 nm) at pH7.0, whereas HOT_75m4 from the same place in deep-sea have blue absorption maxima (490 nm) at pH7.0^8^. The depth of host living brings the difference between those groups. It is primarily related to light penetration availability upon a proportion of the depth. Surface waters are exposed to white light, whereas deeper ocean is mostly exposed to the blue light. It is due to stronger scattering and absorption of other wavelength light at the upper depth^13^. Also, several amino acids changes on retinal binding pocket trigger dramatic change of absorption maximum^20^. If 105th position on PR (Leu in GPR and Gln in BPR) is changed to each other, their absorption spectra also can be interconverted. To adapt to cold environmental temperature, the photo cycling rate of PRs was modified. NPR8 from the sea near the Korea Arctic Research Station Dasan at NyAlesund, Svalbard, Norway showed a different temperature dependency between GPR and BPR^21^.

Interestingly, only GPR type PRs were found at the Arctic Ocean, but GPR and BPR types were identified on the surface from the Antarctic ocean. For this study, we isolated new BPR type gene from Antarctic Ocean and it was characterized by using several biophysical methods such as light-driven proton pumping assays, and absorption spectroscopy, laser flash-induced photolysis technique.

## Results

### Two PR homologue genes were founded in total genomic DNA samples from sea near research station King SeJong

**Figure 1:**
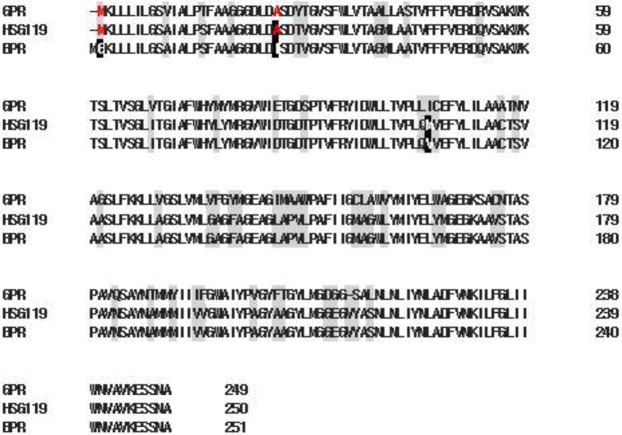
Amino acid sequence alignment of BRP, GRP and HSG119. Shade regions are different position each 3 types of rhodopsin. Inverted characters are different between HSG119 and BPR, but Red ones are the same amino acid with HSG119 and GPR.

Proteorhodopsin homologues genes were amplified from each genomic DNA samples by PCR using conserved primers and degenerate primers (Table S1). The degenerate primers were designed on the basis of the conserved N-terminal and C-terminal regions of previously studies^10,15^. The conserved primers were designed on the conserved regions on helix C and F. We found two PR full length DNA sequences from metagenome sample CKH120(CKH070120-01) and HSG119(HSG070119-03). Genes from CKH120 are perfectly identical to GPR. However, the genes from HSG119 showed several differences on primary sequence. HSG119 showed 99% amino acid sequence homology to BPR and 80% to GPR. Along the amino acid sequence alignment, HSG119 has 3 different positions to BPR (Fig. 1).

### Photochemical properties of HSG119 from antarctic ocean

**Figure 2:**
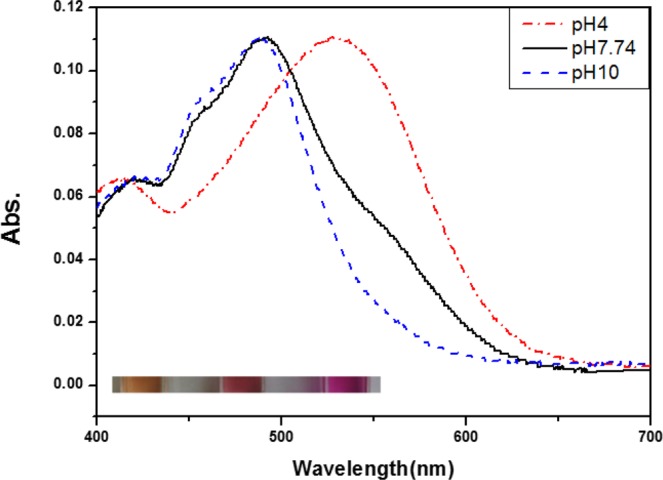
Absorption spectra of HSG119 at various pH. λmax of HSG119 is 535 nm at pH 4, 493 nm at pH 7.74 and 490 nm at pH 10. Each color of HSG119 at different pH shows at the bottom of the spectrum (from left pH 4, pH 7.74, pH 10). Absorption peak of HSG119 has left and right shoulders at pH 7.74. Only left shoulder remain at the alkalic pH. On the other hand, there is no shoulder at acidic pH.

**Figure 3:**
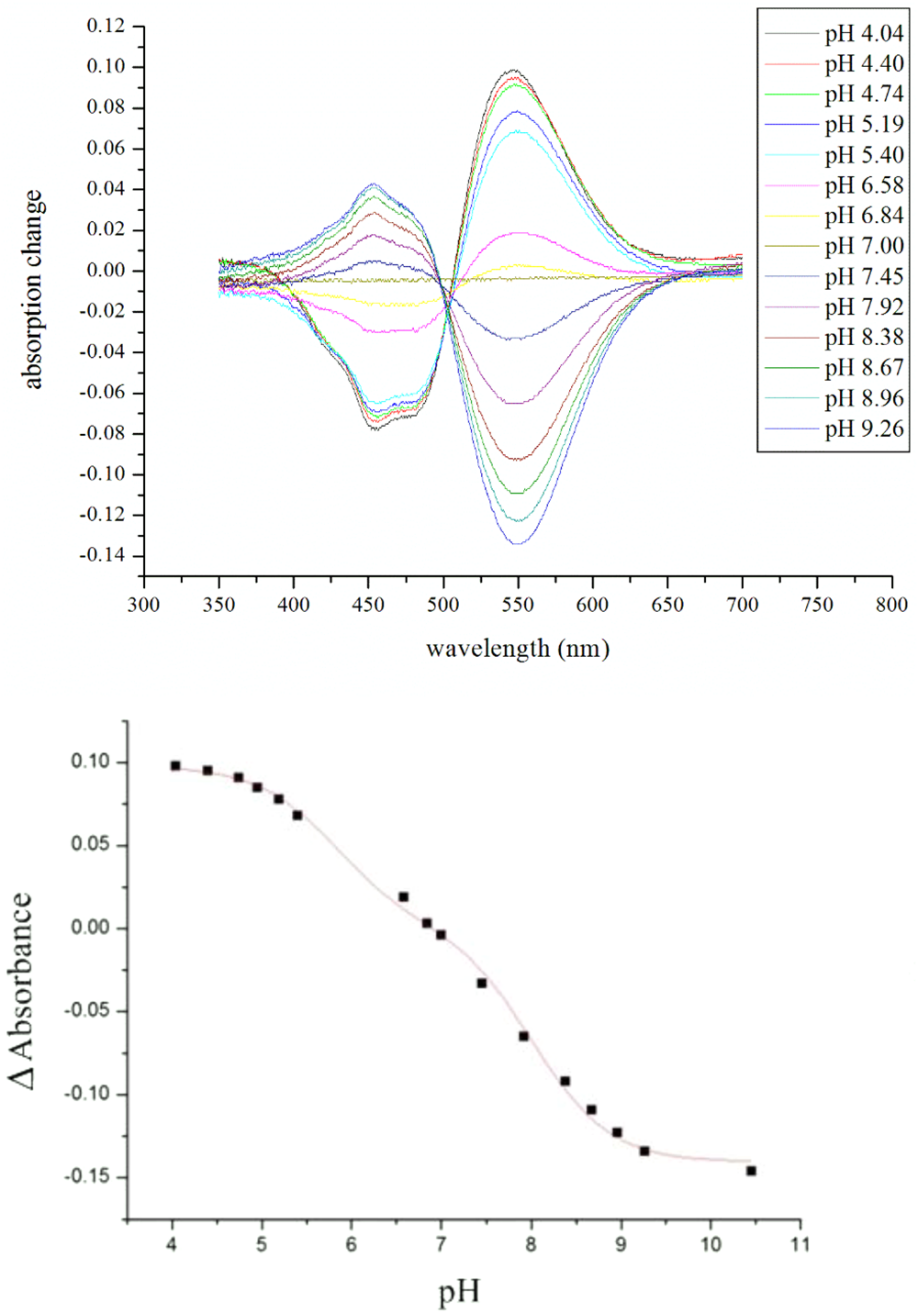
Spectroscopic titration of the Schiff base counterion of HSG119. Absorption spectra were collected at several different pH and compared with absorption spectrum of HSG119 at pH 7. One isosbestic point is identified around 500 nm. pH titration of HSG119 was performed in 50 mM Tris-HCl, 150 mM NaCl, 0.02% DDM. HSG119 has the two pKa of around 5.8 and 8.0. Absorbance difference at several pH were collected at 550 nm wavelength.

**Figure 4:**
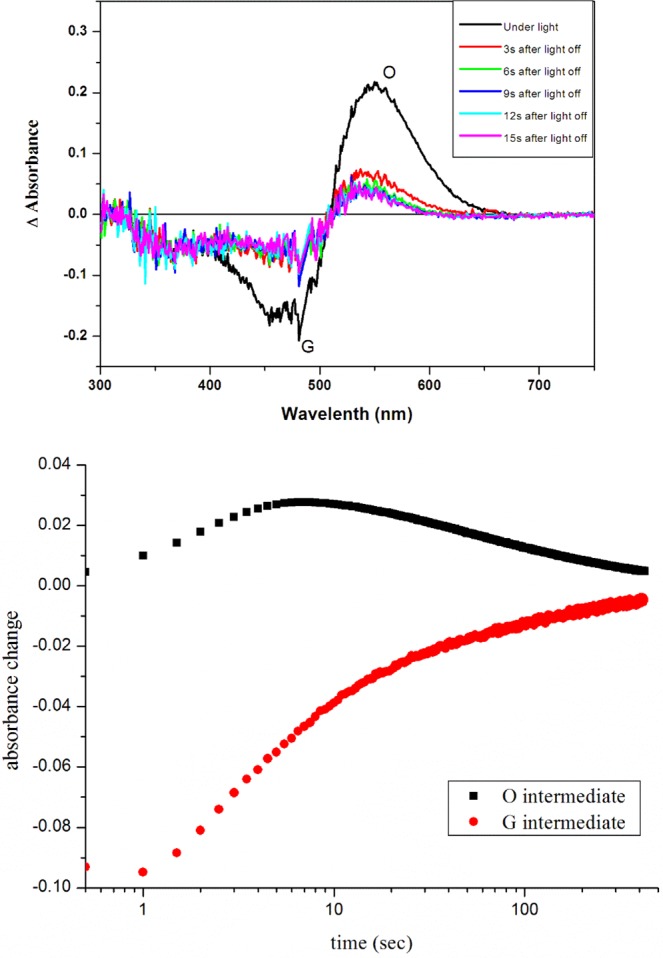
Time-resolved difference spectra and photocycling rate of HSG119. Absorption spectra (upper panel) were collected every 3 second after light illumination until 15 second. Clearly O intermediate was produced, but M intermediate was not detected. We measured rise and decay rate of ground and O intermediate states (bottom panel).

Absorption maxima of HSG119 rhodopsin is 535 nm, 493 nm, and 490 nm at pH 4, 7 and 10, respectively (Fig. 2). It is very similar spectra to that of BPR. But, there are some shoulders on both side of HSG119 rhodopsin spectrum at pH 7. When the proteins are under pH10, right side shoulder is disappeared but left side shoulder is still existed. Both shoulders are disappeared if the proteins are under pH 4. Absorption spectrum is shifted by the proton concentration. Especially, it showed large red shift (39 nm) at pH 4 versus spectrum at pH 7. We collected absorption spectra of HSG119 rhodopsin under various pH conditions. We could titrate the Schiff base counter-ion of HSG119 rhodopsin from spectra under various pH conditions. Only one isosbestic point is shown at around 500 nm wavelength (Fig. 3 upper picture). We used absorption maximum at the 550 nm and set spectrum of pH 7 as a reference for calculation. After the calculation, we could get the pKa value of HSG119 rhodopsin around 5.8 and 8.0. Observation of two pKa is similar feature of previous reported BPR (pKa at 6.2 and 7.8)* (Fig. 3 lower picture). Before functional study for HSG119 rhodopsin, we measured photocycling rate for measuring photointermedates. First, we could detect O intermediates by the time-resolved difference spectra measurement (Fig. 4). There is negative direction spectrum change between 450 and 490 nm wavelength when protein got light activation. It is broad, but the absorption spectrum of HSG119 rhodopsin (at pH 10) is near at that range. There is a big positive peak of O intermediate around at 550 nm wavelength. The amplitude of peak is very similar as negative peak around at 480 nm by decrease of ground state. We measured the spectral changes for 15 sec after light off. M intermediate of HSG119 could not be measured and O decay is slower than GPR and BPR, but faster than NPR8 that contains Y200N mutation^21^.

### Temperature dependency of HSG119 rhodopsin from antarctic ocean

**Figure 5:**
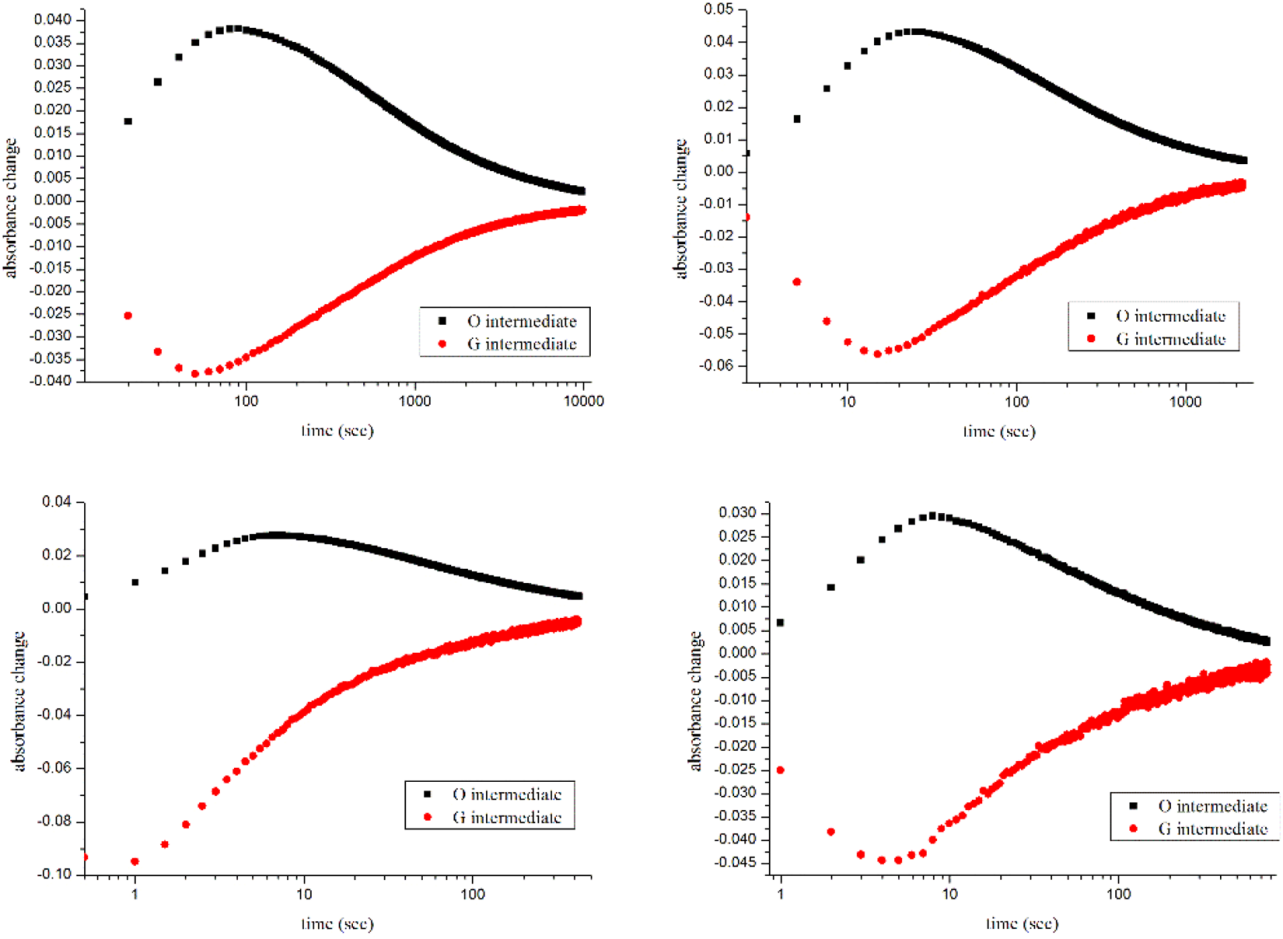
The decay rate of the photointermedates of HSG119 rhodopsin by the temperature. The rate of photochemical reaction of HSG119 rhodopsin at 5 °C, 15 °C, 25 °C and 35 °C(from top-left, top-right, bottom-left and bottom-right, respectively). Temperature and photocycling rate of HSG119 rhodopsin has a positive-relationship.

**Figure 6:**
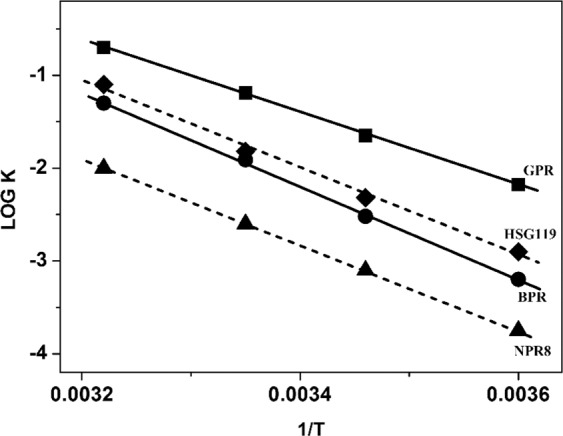
Temperature dependence of the rate of the O decay in GPR, BPR, NPR8 (Y200N) and HSG119. We compared dependency of the O decay rate of HSG119 with BPR, GPR and NPR8. The slope of each rhodopsin is −4430(GPR), −4716(HSG119), −5433(BPR) and −5082(NPR8). NPR8 is from previously study about Y200N mutant type of PR discovered from Arctic ocean^21^. R^2^ is over the 99%, R^2^ of HSG119 shows on the figure.

We measured photocycling rate of HSG119 rhodopsins using various temperature. We focused on temperature dependency of O decay of HSG119. We set the temperature conditions as 5 °C, 15 °C, 25 °C and 35 °C(Fig. 5). HSG119 rhodopsin shows positive relationship on temperature and photocycling rate. We compared temperature dependency with GPR, BPR and NPR8 (Y200N) based on the slopes of the Arrhenius plot (Fig. 6). NPR8 was well-studied in previously report, the slope of NPR8 is in the middle between those of GPR and BPR.. Temperature dependency of HSG119 is similar to that of NPR8 (Y200N) than that of GPR and BPR^21^. When temperature drops, the photocycling rate of O intermediates of HSG119 also decreased down

### Proton pumping activity of HSG119 rhodopsin

**Figure 7:**
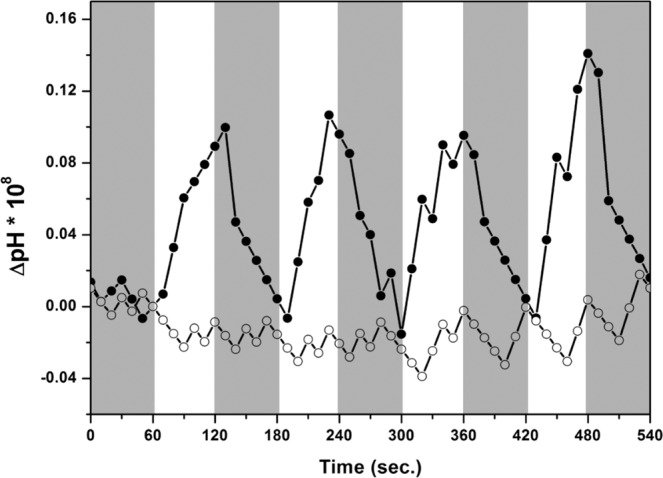
Proton pumping activity of HSG119. We measured proton pumping activity of HSG119 using sphaeroplasts. After one minute for dark adaptation (not shown), one minute for dark and one minute for white light illumination were repeated for 4 times. Closed circle is HSG119 rhodopsin +10uM CCCP and open circle is only HSG119 rhodopsin.

For the functional study, we measured proton pumping ability (Fig. 7). We made sphaeroplast of HSG119 expressed cell. First, we put sphaeroplast at dark condition for 1 min. After dark adaptation, we measured the pH changes of 1 min duration of dark and light illumination. HSG119 rhodopsin clearly showed outward proton transfer during light activation. It is pretty good efficiency compared to almost 60% level of GPR.

### Optical property of HSG119 mutants, D97N and E108Q

**Figure 8:**
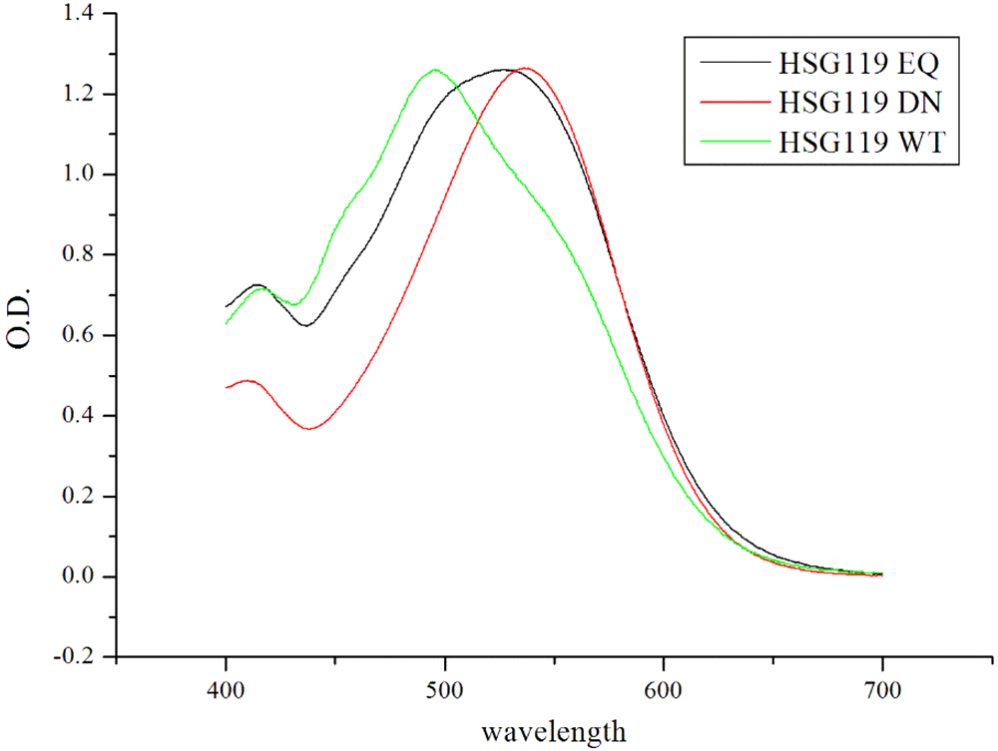
Absorption spectra of HSG119 mutants. λmax of HSG119 D97N and E108Q were 535 nm and 529 nm, respectively, at pH 7. The D97N mutant does not have the shoulders (see red line) and E108Q mutant lose only right shoulder (see black line) on the spectrum.

Two important residues of HSG119 rhodopsin are verified by characterization of mutants - the proton acceptor Asp97 and donor Glu108, respectively. We constructed D97N and E108Q mutants. We checked absorption spectra for each mutant. Both D97N and E108Q showed large red shift compare to WT of HSG119 rhodopsin. Each mutant absorbs light maximally at 535 nm (D97N HSG119) and 529 nm (E108Q HSG119). Shoulders on both side of WT were no longer existed in D97N mutant. Otherwise, E108Q still has broad shoulder on left side which of the amplitude is larger than WT. But right shoulder was disappeared (Fig. 8).

## Discussion

PRs from Arctic and Antarctic Ocean are well-known in terms of their sequences and absorption maxima^8,13,14,22^. In the previous study, PalE6 (southern ocean Palmer station: PAL clone) that was reported from the Antarctic Ocean absorbs blue light (λmax = 490 nm, pH 7). However, most of the PR variants in the Arctic Ocean absorb green light (λmax = 527 nm, pH 7). Their amino acid sequences are similar to that of GPR from Monterey Bay Proteorhodopsin^8^. Based on the previous report, the color of PRs has a small influence on ion transport activity^21^.

In this study, we found BPR homologue HSG119 rhodopsin from the Antarctic Ocean. HSG119 rhodopsin has three different changes upon BPR. HSG119 has maximum absorption at 493 nm at pH 7 that is similar to BPR, but it did not show the typical spectrum shape of type I rhodopsin. HSG119 rhodopsin has two shoulders on both sides of the spectrum. These shoulders make HSG119 absorbs light broadly and not only blue light but also green light region. Compared with BPR, GPR and HSG119 rhodopsin at 525 nm, absorption maxima of GPR, HSG119 rhodopsin absorbs light above 80% of GPR and also over 10% than those of BPR. We constructed two mutants D97N and E108Q, those were mutations of proton acceptor and donor, respectively. At D97N mutant lost its shoulders and therefore it is proved that the protonation of proton accepter is involved in its shoulders. Probably, these shoulders were influenced by the interaction between the negative charge of Asp97 and chromophore, retinal. Also, HSG119 rhodopsin has methionine at 106 position, but BPR has valine and this position is a nearby retinal binding site that might involve in color-tuning.

Previously, it is suggested that PRs could be divided based on the photocycling rate rather than absorption maxima because the absorption peak of PRs has little relationship with their function, proton transport. NPR8 showed the dependency on temperature between those of GPR and BPR. We have reported that Arctic PRs have adapted to environmental temperature changes^21^. Here we showed that the temperature dependency of HSG119 rhodopsin was similar to NPR8 as expected. The big difference of HSG119 rhodopsin compared to NPR8 is proton pumping activity. HSG119 has good proton pumping activity but NPR8 does not has it. HSG119 rhodopsin could chase two hares called cold adaptation and proton pumping activity at once and successfully adapted.

The result showed on cold adaptation of Arctic PRs cannot be answered for the distribution of PRs. The color of PRs has little related to cold temperature adaptation. Here, we suggested the answer for why the BPR like rhodopsin existed on the surface of Antartic Ocean. Metagenome samples were collected from Marian Cove, inside of Maxwell Bay. The glacial wall of this area collapses very often. Also, deep ocean heat melting the Antarctica’s ice. Because of that, sediment, many organic nutrients and materials are mixed easily and often in this area. This phenomenon caused the surface of the sea to be quite turbid^23^. And the range of tide in this area is 1,800 mm. It makes frequent seawater currents. Rising current also can be generated and it caused the migration of middle or deep-sea microbe to the surface of the sea. These might be able to make the finding of BPR like rhodopsins at the sea surface area.

Briefly, these results suggested that the protein activity is modified and adapted if environmental conditions are changed.

## Methods

### Collection of samples and extraction of DNA from the marine bacteria

The Antarctic samples were collected from sea surface from the region around King Sejong Station at King George Island (62°13.208′S – 58°47.114‘W), Antarctica (Table S2). Water temperature was 0.9 °C, pH was 7.74, and salinity was 32.6‰. Two liters of seawater were concentrated using 0.2 μm Sterivex filters (Millipore) and transported to the laboratory in Korea at −20 °C. Total genomic DNA was extracted using a modified CTAB method as described in the previous study^24^.

### PCR amplification from the DNA samples from antarctic ocean

Metagenome samples used as a template for PCR to discover new types of proteorhodopsins. Primers were designed using conserved N-terminal and C-terminal regions of MBP (eBAC31A08) and using conserved regions in Helix C and F. Non-degenerate primer, generate primer (non-degenerate primer, 5′-ATGAAANNATTANTGATNTT-3′, generate primer, 5′-ATGAAATTATTACTGATATTAGG-3′, reverse primer, 5′-AGCATTAGAAGATTCTTTAACAGC-3′) and conserved primer (conserved primer, 5′-TTNMGNTAYATHGAYTGG-3′, reverse conserved primer, 5′-CGGGTAAATCGCCCAACC-3′) were used^10,15^. PCR was performed for 35 cycles at 95 °C for 2 min, 47~51 °C for 2 min 30 sec, 72 °C for 2 min 30 sec. The proteo-opsin genes were amplified with *Taq* polymese (Vivagen Korea) and cloned into T-bunt vector (Solgent, Korea)

### Expression and purification of proteorhodopsin-like new rhodopsin, HSG119

pKA001 plasmid contains a proteorhodopsin gene and a mouse dioxygenase gene which can convert β-carotene to two all-trans retinal^25^. All PR variants were cloned into pKA001 and were expressed in *Escherichia coli* strain UT5600 using retinal. PR genes are under the lacUV5 promoter and mouse dioxygenase genes are under the PBAD promoter to produce dioxygenase in *E*. *coli*. For photochemical measurements, UT5600 cells transformed with rhodopsin cloned pKA001 were induced with 1 mM IPTG (Isopropyl β-D-1-thiogalactopyranoside) and 5~10 uM all-trans retinal (Sigma, USA) for 4~8 hrs at 37 °C. Rhodopsin expressed cells were resuspended with sonication buffer (150 mM NaCl, 50 mM Tris-HCl, pH7.0), sonicated for 4 min by 15 sec pulse (Branson sonifier 250), and the membrane fraction was treated 1% n-dodecyl-α-D-maltopyranoside(DDM) (Anatrace, USA). The solubilized fraction was incubated with Ni^2+^-NTA agarose (Qiagen, USA) and protein was eluted with 250 mM Tris-HCl (pH7.0), 150 mM NaCl, and 0.02% DDM.

### Measurement of the absorption spectra and pKas

The setting for absorption spectroscopy to measure absorption maxima of the the rhodopsin and pKas of the Schiff base counterion in purified PRs is followed to previous research^21^. The absorption spectra were recorded with UV/VIS spectrophotometer (UV-2550, Shimadzu, Japan) at pH 4, pH 7.74, and pH 10. In order to calculate the pKas of the primary proton acceptor, performed the pH titration in the range of pH 4 ~ pH 10 and the spectrum at pH 7.0 was used as a reference. Collected data from different absorption spectra were determined and fitted with functions containing pKa components[y = A/(1 + 10 ^pH-pKa^)] using Origin Pro7.0^26^.

### Preparation of the sphaeroplast and proton pumping measurements

To measure the pumping activity, the sphaeroplast of new isolated PR is prepared similar to previous research^21,26^. Rhodopsin-expressed cells (250 mL) were centrifuged and suspended in 10 mL of plasmolysis buffer (30 mM Tris-HCl, pH 8.0, and 20% sucrose) with lysozyme (Usb, USA). Sphaeroplasts were collected and resuspended in 400 uL DNA lysis buffer (100 mM KPi, pH 7.0, 20 mM MgSO_4_, 20% sucrose, 1.6 mg DNase I) and injected using a 1 mL syringe (26 gauge needle) into 200 mL of rapidly shaking osmotic shock solution (50 mM KPi, pH 7.0). After shaking at 37 °C for 15 min, Na-EDTA was added to a final concentration of 10 mM and shaked for 15 min, MgSO_4_ was added to a final concentration of 15 mM and shaked for another 15 min. Sphaeroplast vesicles were collected at 30,778 × g for 1 hr at 4 °C (Beckman XL-90 ultracentrifuge, USA) and washed with 10 mL unbuffered solution (10 mM NaCl, 10 mM MgSO_4,_ 10 uM CaCl_2_). Finally, sphaeroplasts were resuspended in 3 mL of unbuffered solution^26^. Samples were illuminated through the short wave cut-off filter (>440 nm, Sigma Koki SCF-50S-44Y, Japan) in combination of focusing convex lens and heat-protecting (CuSO_4_) filter. The pH values were monitored (Horiba pH meter F-51) and pH data were transferred and recorded automatically with Horiba data Navi program. Starting pH was pH 8.2. After one minute for dark adaptation, one minute for dark and one minute for white light illumination were repeated for 4 times.

### Time-resolved difference spectra and laser induced absorption difference spectroscopy

Samples were illuminated through Cole-Parmer illuminator (41720 series) and time-resolved difference spectra were measured by S-3100 diode-array spectrophotometer (Shinco, Korea). Flash-induced absorbance changes were measured on RSM 1000 (Olis, USA) and laser flash was from Nd-TAG pulse laser (Contiuum, Minilight II, 532 nm, 6 ns, 25 mJ)^2,21,27^. 10–30 signals were averaged for measuring the rate of formation and decay of the photointermediates. PR expressed *E*. *coli* membrane fractions suspended with 0.08% DDM in sonication buffer. Heavy membranes or cell debris were eliminated by centrifugation at 4,000 × g for 4 min. Light membrane were collected by ultracentrifugation at 40,000 × g for 20 min and then resuspended in DIW. The membranes were embedded into 7% polyacrylamide gels which were soaked in 150 mM NaCl at pH9.0 for measuring laser-induces absorbance difference kinetics of PR^27^.

## Supplementary information

Supplementary Information.
