# Supplementary material for: Temperature Dependency of Proton Pumping Activity for Marine Microbial Rhodopsin from Antartic Ocean
Source: Sci Rep. 2020 Jan 28;10:1356. doi: 10.1038/s41598-020-58023-5 (PMC6987182; doi:10.1038/s41598-020-58023-5)
Supplement: Supplementary file 1 — Supplementary Information. [file 41598_2020_58023_MOESM1_ESM.docx]

Table S1. Used Primers for PCR of genomic DNA samples.

| PRIMER NAME | | PRIMER SEQUENCE |
| --- | --- | --- |
| FORWARD | Degenerate | 5’-ATGAAATTATTACTGATATTAGG-3’ |
|  | Non-Degenerate | 5’-ATGAAANNATTANTGATNTT-3’ |
|  | Conserved  (FRYIDW) | 5’-TTNMGNTAYATHGAYTGG-3’ |
|  | Conserved  (LRYVDWILT) | 5’-CTCCGTTATGTTGATTGGATTTTAACA-3’ |
| REVERSE | Non-Degenerate | 5’-AGCATTAGAAGATTCTTTAACAGC-3’ |
|  | Conserved  (GWAIYP-rev) | 5’-CGGGTAAATCGCCCAACC-3’ |
|  | Conserved  (WFLLVGWAIYP-rev) | 5’-CGGGTAAATCGCCCAACCAACTAGAAGGAACCA-3’ |

Table S2. Genomic DNA samples at collection sites

| SAMPLE NAME | LOCATION |
| --- | --- |
| BS-142-SWSV-9 | Bering sea |
| BS-143-SWSV-9 |  |
| CKH070119-01 | Dockside nearby King Sejong Antarctic Station  (from biofilm) |
| CKH070120-01 |  |
| HSG070114-06 | Coast nearby King Sejong Antarctic Station  (from sea water) |
| HSG070119-03 |  |
